# A Phase 1/2 Study of Flavocoxid, an Oral NF-κB Inhibitor, in Duchenne Muscular Dystrophy

**DOI:** 10.3390/brainsci11010115

**Published:** 2021-01-16

**Authors:** Gian Luca Vita, Maria Sframeli, Norma Licata, Alessandra Bitto, Sara Romeo, Francesca Frisone, Annamaria Ciranni, Giovanni Pallio, Federica Mannino, M’Hammed Aguennouz, Carmelo Rodolico, Francesco Squadrito, Antonio Toscano, Sonia Messina, Giuseppe Vita

**Affiliations:** 1 Nemo Sud Clinical Centre for Neuromuscular Disorders, Messina University Hospital, Via C. Valeria 1, 98125 Messina, Italy; gianlucavita81@gmail.com (G.L.V.); mariasframeli@hotmail.it (M.S.); sonia.messina@unime.it (S.M.); 2 Department of Clinical and Experimental Medicine, University of Messina, Via C. Valeria 1, 98125 Messina, Italy; normalicata@hotmail.com (N.L.); abitto@unime.it (A.B.); romeosara@gmail.com (S.R.); francescafrisone@hotmail.it (F.F.); annamaria.ciranni@unime.it (A.C.); gpallio@unime.it (G.P.); fmannino@unime.it (F.M.); aguenoz.mhommed@unime.it (M.A.); crodolico@unime.it (C.R.); francesco.squadrito@unime.it (F.S.); antonio.toscano@unime.it (A.T.); 3 Faculty of Medicine, Mohammed VI University of Health Sciences, Cheikh Khalifa Ibn Zayed Hospital, Bd. Mohammed Taïeb Naciri, Commune Hay Hassani, Casablanca 82 403, Morocco

**Keywords:** Duchenne muscular dystrophy, phase 1/2 study, flavocoxid, NF-κB inhibitor, antioxidant, anti-inflammatory agent

## Abstract

Flavocoxid is a blended extract containing baicalin and catechin with potent antioxidant and anti-inflammatory properties due to the inhibition of the cyclooxygenase (COX) and 5-lipoxygenase (5-LOX) enzymes, nuclear factor-κB (NF-κB), tumor necrosis factor (TNF)-alpha, and the mitogen-activated protein kinases (MAPKs) pathways. This phase 1/2 study was designed to assess the safety and tolerability of flavocoxid in patients with Duchenne muscular dystrophy (DMD). Thirty-four patients were recruited: 17 were treated with flavocoxid at an oral dose of 250 or 500 mg, according to body weight, for one year; 17 did not receive flavocoxid and served as controls. The treatment was well tolerated and nobody dropped out. Flavocoxid induced a significant reduction in serum interleukin (IL)-1 beta and TNF-alpha only in the group of DMD boys on add-on therapy (flavocoxid added to steroids for at least six months). The decrease in IL-1 beta was higher in younger boys. The serum H_2_O_2_ concentrations significantly decreased in patients treated with flavocoxid alone with a secondary reduction of serum glutathione peroxidase (GPx) levels, especially in younger boys. The exploratory outcome measures failed to show significant effects but there was a trend showing that the younger boys who received treatment were faster at performing the Gowers’ maneuver, while the older boys who received treatment were faster at doing the 10-m walk test (10MWT). Therefore, a double-blind, placebo-controlled study for at least two/three years is warranted to verify flavocoxid as a steroid substitute or as add-on therapy to steroids.

## 1. Introduction

Duchenne muscular dystrophy (DMD) is a severe progressive disease that is a result of protein dystrophin deficiency. Deletions, duplications, and point mutations in the DMD gene stop the synthesis of full-length dystrophin in the skeletal muscle and heart, as well as protein variants in other tissues [1,2]. In muscles, the link between the extracellular matrix and the cytoskeleton is perturbed, leading to instable muscle membranes and cell necrosis, followed by inefficient regeneration [3]. Treatment options for DMD are still limited. Steroids are recommended in any type of DMD gene mutation, since they improve muscle strength and function for up to two years. Ataluren, the first approved drug for DMD, can be prescribed to patients with premature stop codon mutations. Exon-skipping technology is still under investigation in an attempt to induce cellular machinery to “skip over” an aimed exon and to restore the reading frame, so that a truncated but partially functional dystrophin protein is produced [4,5]. Although eteplirsen, golodirsen, and viltolarsen have received accelerated approval from the U.S. Food and Drug Administration (FDA), requiring a confirmatory study on the increased level of dystrophin expression and their clinical benefit [6,7,8], the development of other exon-skipping drugs such as suvodirsen was recently stopped because of the failure to increase dystrophin levels (presented at the 2020 Muscular Dystrophy Association Conference).

Cell necrosis in DMD muscles parallels a strong inflammatory response involving different factors such as mitogen-activated protein kinases (MAPKs), cyclooxygenase (COX), 5-lipoxygenase (5-LOX), leukotriene B-4, tumor necrosis factor-alpha (TNF-alpha), reactive oxygen intermediates (ROI), nuclear factor-κB (NF-κB), and Hippo signaling pathways, all of which are considered possible therapeutic targets [9,10,11,12,13]. The transcription factor NF-κB, which serves as a main mediator of inflammatory responses, is over-expressed in DMD muscles [14] and its inhibition by different pharmacological compounds or procedures has beneficial effects on the functional, biochemical, and morphological aspects of the muscles and hearts of *mdx* mice, which is the murine model of DMD [15,16,17,18,19,20,21].

Flavocoxid is a medical food comprising plant-derived flavonoids with anti-inflammatory activity and is used to provide benefit to people with chronic osteoarthritis [22]. It contains standardized flavonoids, baicalin, and catechin. Baicalin belongs to a class of flavonoids knows as free-B-ring flavonoids from the root of *Scutellaria baicalensis* (Chinese skullcap), whereas catechin is a flavan from *Acacia catechu* (black catechu) [23]. Flavocoxid has potent antioxidant action through the inhibition of the COX and 5-LOX enzymes to deliver anti-inflammation activity [24,25]. Moreover, flavonoids inhibit the NF-κB, TNF-alpha, and MAPKs pathways through their antioxidant properties [26,27]. Our group demonstrated that flavocoxid has similar beneficial effects to methylprednisolone in *mdx* mice. Both compounds improve the functional muscular properties in vivo and ex vivo and inhibit the COX/5-LOX and MAPKs pathways, whereas NF-κB activity is dampened more efficiently by flavocoxid treatment, probably due to its additional antioxidant effect. Moreover, flavocoxid is more effective than methylprednisolone in reducing muscle necrosis and enhancing muscle regeneration [28]. All of this evidence supports the hypothesis that flavocoxid, with its multi-target effect, may be an appropriate pharmacological approach to reduce muscle damage in dystrophinopathy.

The aim of this phase 1/2 study was to assess the safety and tolerability of flavocoxid with secondary endpoints to demonstrate the biological activity by laboratory assays and exploratory outcome measures in patients with DMD.

## 2. Patients and Methods

This was a pilot, open-label, single-center, proof-of-concept study of flavocoxid administered for one year in DMD patients, alone or in association with steroids (ClinicalTrials.gov Identifier: NCT01335295). Flavocoxid (Limbrel^®^) was a kind gift from Primus Pharmaceuticals, Inc. (Scottsdale, AZ, USA). The trial project was presented at and reviewed by the TREAT-NMD Advisory Committee for Therapeutics (TACT). Ethical approval was obtained from the local ethics committee (Comitato Etico Scientifico, Azienda Ospedaliera Universitaria Policlinico “G. Martino”, Messina, Italy, 5/2011) and the patients’ legally authorized representative (LAR) provided written informed consent.

### 2.1. Inclusion Criteria

The inclusion criteria were as follows: DMD diagnosis, confirmed by muscle biopsy and molecular analysis via multiplex ligation-dependent probe amplification (MLPA) assay; age range between 4 and 12 years; unaided ambulation for at least 75 m, unassisted during the six-minute walk test (6MWT); patients able to perform all evaluation tests in a 12-month period; since steroids are usually prescribed in DMD to help maintain muscle strength, patients not taking steroids or on steroid therapy for at least six months; LAR able to understand and provide informed consent; written informed consent signed by LAR.

### 2.2. Exclusion Criteria

Excluded were patients treated with other drug analogues, similar to or interacting with flavocoxid, or immunosuppressive therapy (other than steroids) within three months prior to the start of the study protocol; participation in another study with an investigational drug or supplements within three months prior to the start of the study protocol; existence of cognitive impairment influencing the performance of the assessment tests; history of major surgery within 30 days prior to the start of the study protocol; expectation of a major surgical procedure (e.g., scoliosis surgery) during the 12-month treatment period of the study; current participation in any other therapeutic clinical study; need of daytime ventilator assistance; presence of liver diseases or assumption of any hepatotoxic agent; routine laboratory values out of the normal ranges if clinically meaningful; prior or ongoing medical condition (e.g., concomitant illness, behavioral disorder, or psychiatric condition), medical history, physical findings, electrocardiogram findings, or laboratory abnormalities that, in the investigator’s opinion, could adversely affect the safety of the subject, making it unlikely that the course of treatment or follow-up would be completed, or that could impair the evaluation of the study results.

### 2.3. Randomization

Patients were allocated to two study groups, namely, the flavocoxid-treated or untreated groups, according to a mixed randomization scheme with an unbalanced starting block of five patients and subsequent randomly permuted balanced blocks of two or four patients [29].

### 2.4. Biological Activity

The biological activity of flavocoxid was assessed in treated patients by measuring the serum levels of interleukin 1 beta (IL-1-beta), TNF-alpha, hydrogen peroxide (H_2_O_2_), and glutathione peroxidase (GPx). Briefly, for the IL-1-beta and TNF-alpha assays, ELISA Kits (IL-1-beta Human ELISA Kit #BMS224-2 and TNF-alpha Human ELISA Kit #KHC3011, respectively) were purchased from Invitrogen Thermo Fisher Scientific, Monza, Italy. Serum aliquots of 50 μL were used for the single determination following the procedure steps of the assay. The H_2_O_2_ assay was performed in serum aliquots of 200 μL by spectrophotometry using an Amplex Red Hydrogen Peroxide/Peroxidase Assay Kit (#A22188, Molecular Probes Inc., Eugene, OR, USA). The GPx assay was conducted in serum aliquots of 100 μL using an ELISA Kit (#353919, Calbiochem, Darmstadt, Germany). Each determination was performed in triplicate.

### 2.5. Endpoints

The primary endpoints of this study were the safety and tolerability of flavocoxid, which were assessed through the symptoms and signs reported by the patients and relatives, as well as by physical examinations, vital signs measurements, blood tests, and cardiac and pulmonary evaluations. The latter two were performed by the same examiner. All adverse events and laboratory abnormalities were detected.

The secondary endpoints were the following functional assessments:

- North Star Ambulatory Assessment (NSAA), which is a 17-item scale, ranging from standing (item 1) to running (item 17) and including several items assessing abilities that are necessary to remain functionally ambulant, with a total score ranging from 0 if all the activities are failed to 34 if all the activities are achieved [30].
- The timed items are included in NSAA, i.e., the time to rise from the floor (Gowers’ time) and the 10-m walk test (10MWT) [30].
- The 6MWT, performed according to the established guidelines [31]. The evaluator instructed the patient to walk at his own pace while covering as much distance as possible during the allotted time. During the test, the patient was allowed to rest or stop and then continue as soon as he could resume walking. At the end of the six minutes, the distance covered was recorded. The 6MWT was performed along a long, flat, straight, corridor with turnaround points at an interval of 25 m.
- Forced vital capacity (FVC), measured using a standard spirometer, taking the best of three attempts [32].

### 2.6. Statistical Analysis

An intention-to-treat analysis was performed using the last observation in the case of missing data. Data from all enrolled patients were included in the statistical analysis. Statistical analysis was performed using GraphPad Prism, version 8.4.3.00 (GraphPad Software, La Jolla, CA, USA). A comparison between measures was performed using the Mann–Whitney test and the Wilcoxon matched-pairs signed rank test. The Friedman test was used to compare measurements at baseline (T0), after six months (T6), and after 12 months (T12). The results are expressed as mean ± standard deviation (SD) or standard error of the mean (SEM). A level of significance of *p* < 0.05 was considered.

## 3. Results

**Figure 1:**
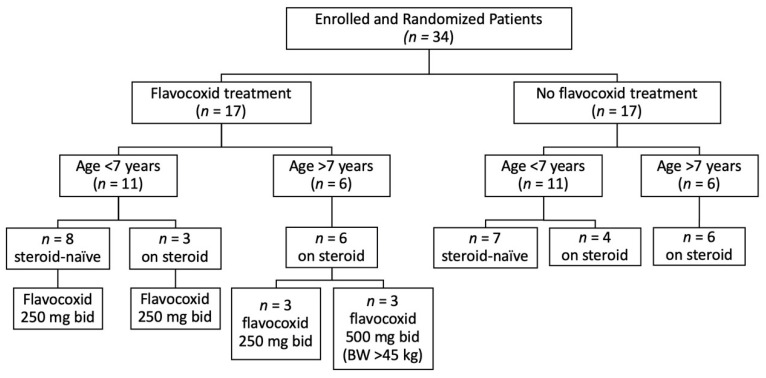
Study profile. All patients completed the study. BW, body weight.

A total of 34 DMD boys were enrolled. Seventeen patients, aged 4–12 years, received flavocoxid at an oral dose of 250 mg (*n* = 14 with body weight <45 kg) or 500 mg (*n* = 3 with body weight >45 kg) BID for one year. Seventeen patients, aged 4–12 years, did not receive flavocoxid and were checked as controls. For the subsequent analysis, the treated group was divided into 11 patients younger than 7 years (8 steroids-naïve and 3 on steroids) and 6 patients older than 7 years, all on steroids. The age of 7 was chosen according to our previous evidence that 7 years is the age when DMD boys experience a slope of deterioration [30]. Likewise, the untreated group was divided into 11 patients younger than 7 years (7 steroids-naïve and 4 on steroids) and 6 patients older than 7 years, all on steroids (Figure 1). There was no significant difference between the flavocoxid-treated and untreated groups in any of the baseline characteristics (Table 1).

**Figure 2:**
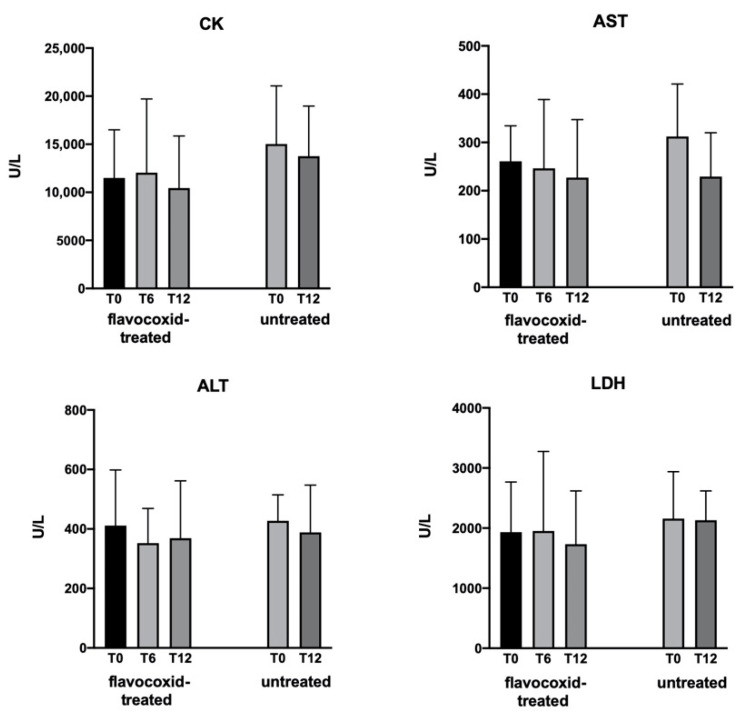
Levels of serum creatine kinase (CK), aspartate aminotransferase (AST), alanine aminotransferase (ALT), and lactate dehydrogenase (LDH) measured in patients at baseline (T0) and after 6 and 12 months (T6 and T12, respectively) of treatment with flavocoxid, and in untreated controls at T0 and T12. Data expressed as mean ± SD. There were no significant differences within groups versus T0.

Flavocoxid was well tolerated and no patients dropped out. The safety assessments, including vital signs and cardiac and pulmonary evaluation, showed no differences between T0, T6, and T12 in the treated group. There were no serious side effects and the adverse events that were considered unrelated to treatment were cough (*n* = 6), diarrhea (*n* = 2), headache (*n* = 1), and fever (*n* = 4). The hematological parameters tested showed no significant differences in the treated and untreated groups. Figure 2 reports the serum concentrations of creatine kinase (CK), aspartate aminotransferase (AST), alanine aminotransferase (ALT), and lactate dehydrogenase (LDH) in the treated and untreated groups with no statistical differences among time points.

**Figure 3:**
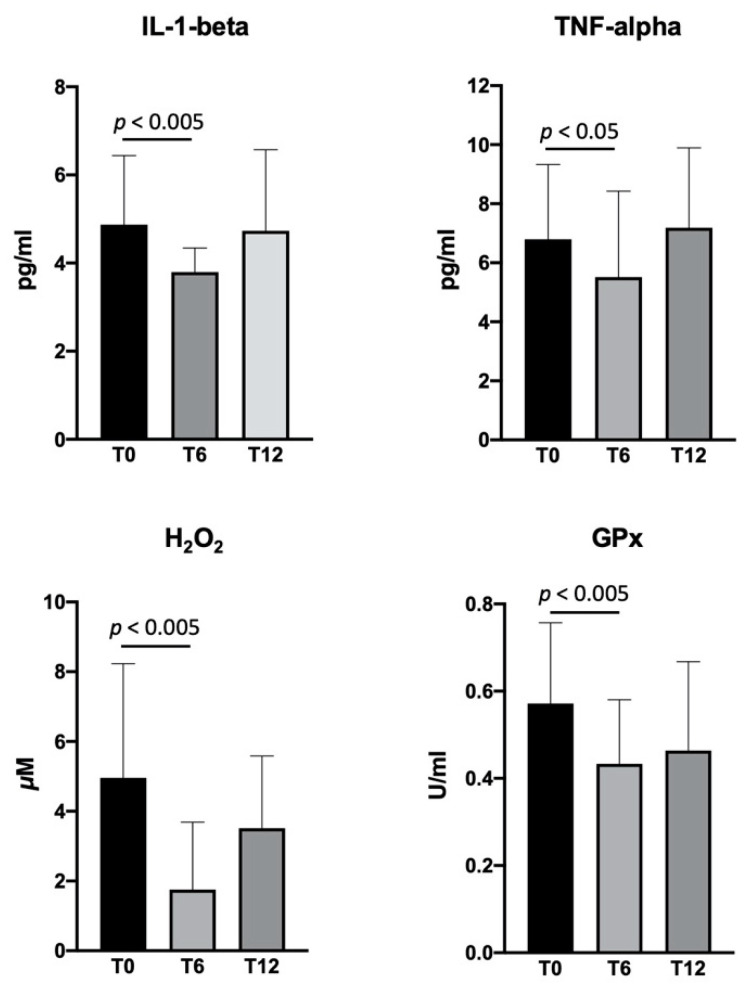
Levels of serum interleukin (IL)-1 beta, tumor necrosis factor (TNF)-alpha, H_2_O_2_, and glutathione peroxidase (GPx) measured in DMD patients (*n* = 17) at baseline (T0) and after 6 (T6) and 12 (T12) months of treatment with flavocoxid. Data are expressed as mean ± SD.

**Figure 4:**
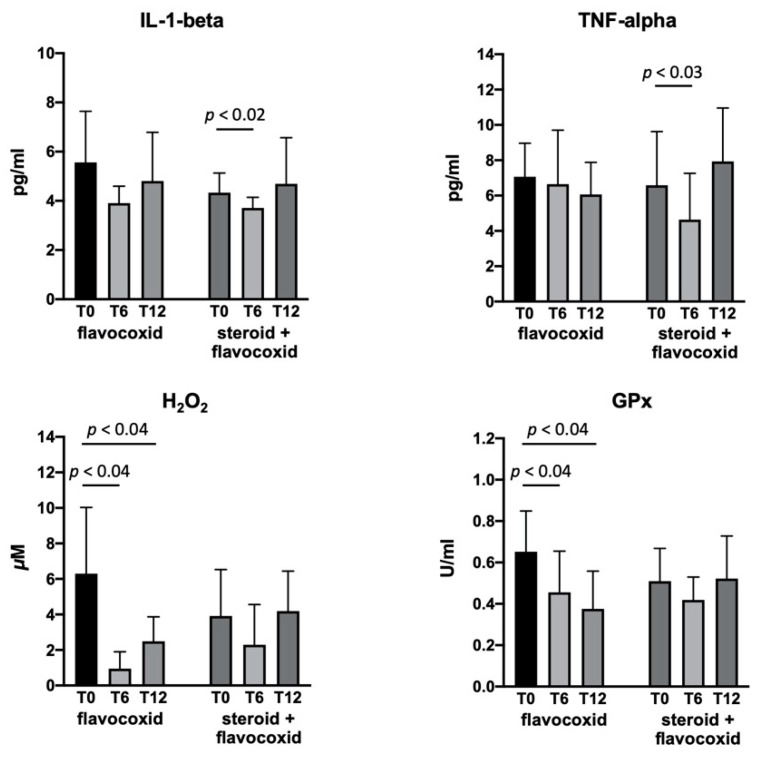
Levels of serum IL-1-beta, TNF-alpha, H_2_O_2_, and GPx measured in patients at baseline (T0) and after 6 (T6) and 12 (T12) months of treatment with flavocoxid. Eight boys were steroids-naïve and were treated with flavocoxid alone; nine boys were already treated with steroids and started flavocoxid as an add-on therapy. Data are expressed as mean ± SD.

**Figure 5:**
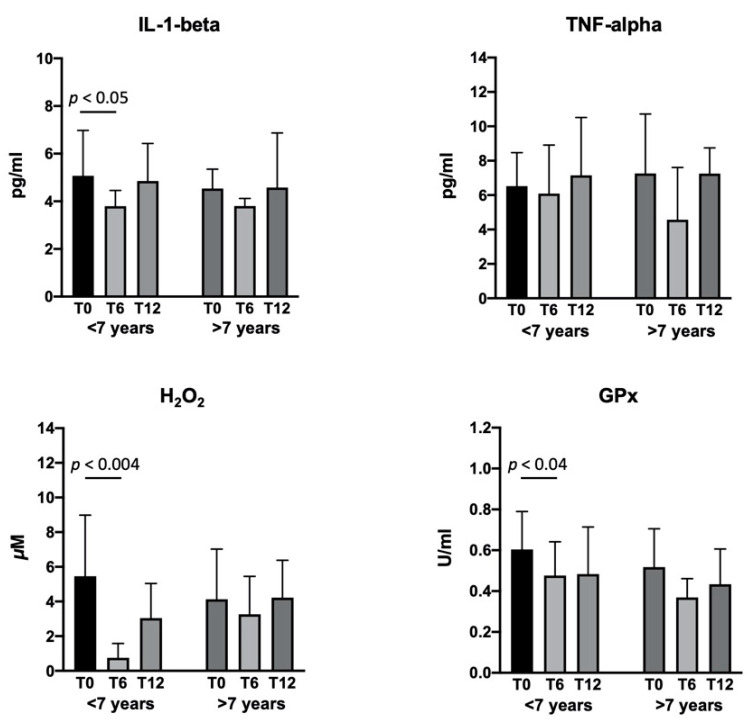
Levels of serum IL-1-beta, TNF-alpha, H_2_O_2_, and GPx measured in patients at baseline (T0) and after 6 (T6) and 12 (T12) months of treatment with flavocoxid. Eleven boys started treatment when aged less than seven years; six boys started flavocoxid when aged greater than seven years. Data are expressed as mean ± SD.

The serum concentrations of IL-1-beta, TNF-alpha, H_2_O_2_, and GPx in the flavocoxid-treated group showed a significant reduction at T6 versus baseline, with no change at T12 (Figure 3). Since some patients started flavocoxid after having already been on steroids for at least six months (*n* = 9), whereas others were steroids-naïve (*n* = 8), we analyzed the serum biomarkers after dividing the patients in two groups. IL-1-beta and TNF-alpha significantly reduced in the DMD patients six months after the addition of flavocoxid to steroid therapy, with a return to baseline values at T12; the patients treated with flavocoxid alone did not show any changes. Flavocoxid treatment induced a significant reduction of the serum values of H_2_O_2_ and GPx at T6 and T12 versus baseline in DMD patients who were steroids-naïve; no significant change was found in the DMD boys treated with steroids after the addition of flavocoxid (Figure 4). To investigate whether an early start of flavocoxid could have a higher effect on the biomarkers, we also allocated treated patients to two different groups: boys aged less than seven years (*n* = 11) and boys aged greater than seven years (*n* = 6). IL-1-beta, H_2_O_2_, and GPx significantly reduced after six months of flavocoxid treatment only in the younger patients (Figure 5). No statistical analysis could be done according to the dosage of flavocoxid used, because only 3/17 received the higher oral dose of 500 mg bid.

Table 2 reports the T12 changes in the measurement of NSAA, Gowers’ time, 10MWT, 6MWT, and FVC. There was significant difference in the treated versus untreated patients, neither when considering all patients together nor when dividing them according to age (i.e., <7 or >7 years). However, a trend toward a higher speed in performing the Gowers’ maneuver in the younger boys who received flavocoxid and the 10MWT in the older boys who received flavocoxid versus the untreated group was evidenced.

## 4. Discussion

Flavocoxid has been marketed in the USA as Limbrel since 2004 as a medical food available only by medical prescription for the dietary management of osteoarthritis. Limbrel has been prescribed mainly by rheumatologists to patients who failed or could not tolerate non-steroidal anti-inflammatory drugs (NSAIDs). It received the overarching designation of “Generally Recognized as Safe” (GRAS) according to scientific demonstration of non-toxicity and safety per U.S. FDA standards. This phase 1/2 study was planned and performed before January 2018, when, upon the FDA’s request, Primus Pharmaceuticals voluntarily recalled all unexpired lots of Limbrel products, since flavocoxid had been associated with a 0.004% incidence of reported hypersensitivity reactions expressed as elevations in serum enzyme levels of a hepatocellular pattern during therapy and with rare instances of clinically apparent liver injury or jaundice and acute hypersensitivity pneumonitis (HP). In investigations when cases were reported, the noted adverse events resolved without residua after terminating product use. At the time of recall, the company announced seeking to work with FDA to return Limbrel to the market as quickly as possible, and thus far, it remains off the market. However, independent medical expert reviews validate that these rare and reversible hypersensitivity reactions were not life threatening. A careful re-evaluation of case reports indicated no common patient or usage patterns except that in serious hepatic cases, patients were often also taking at least one other drug or supplement known to cause hepatic dysfunction. On this basis, a relatively high level of *Scutellaria baicalensis* was administered to 17 patients for over a year, and none manifested clinical symptoms or signs of hepatic dysfunction [33]. Indeed, the same study reported many murine experiments in which *Scutellaria baicalensis* had hepatoprotective actions. Very recently, a post-marketing safety study of new users of flavocoxid (*n* = 3337) versus NSAIDs (*n* = 6674) was completed. It was concluded that the incidence of HP and liver injury linked with flavocoxid was low and slightly increased compared to that with NSAIDs. Flavocoxid users had a significantly lower risk of gastrointestinal bleeding leading to hospitalization. Thus, it was concluded that the risk-benefit profile of flavocoxid may warrant re-evaluation based on these findings [34].

The inflammatory process and oxidative stress downstream of dystrophin deficiency contribute to muscle pathology in DMD, with documented NF-κB activation inducing the expression of several inflammatory mediators. H_2_O_2_, which is one of the molecules generated during oxidative stress, is also a modulator of the NF-κB pathway [35]. Modulation of these events may be critical to the success of dystrophin-replacement therapies [36,37]. Since NF-κB inhibitors have been tested in *mdx* mice as well as in DMD boys [38,39,40] and based on the beneficial effects of flavocoxid in the murine model of DMD, even higher than steroids, as reported by our group [28], we planned a phase 1/2 study. The long-term use of steroids in DMD results in many side effects, including cushingoid appearance, weight gain, excessive hair growth, behavioral abnormalities, osteoporosis, cataract, and an increased risk of bone fractures [41], so that a therapeutically equivalent substance with lesser adverse events could be welcome. In our pediatric DMD cohort, flavocoxid was well tolerated, adverse events related to the treatment were not recorded, and nobody left the study. In addition, serum enzyme abnormalities due to possible muscle and liver damage induced by the treatment were not found.

Since flavocoxid downregulates the gene and/or protein expression of several inflammatory markers and also exerts potent antioxidant activity [42], we investigated its biological activity by measuring exploratory serum biomarkers to assess the effects on inflammation and oxidative stress in DMD boys. NF-κB was measured neither in the muscle nuclear extracts because of ethical reasons, nor in the circulating blood cells by gene expression analysis, but we preferred to analyze NF-κB-dependent markers in the patients’ serum as a simple, easy-to-reproduce, and inexpensive type of measurement. Since steroids have long been part of the care recommendations for DMD patients [4], more than half of our patients in the treated group (and in the untreated group as well) were on steroids for at least six months, and they started flavocoxid as an add-on therapy. Different corticosteroid regimes were used according to the adverse events and patient’s tolerability, since the best schedule of administration has yet to be standardized. We found that flavocoxid induced a significant reduction in serum IL-1-beta and TNF-alpha only in the group of DMD boys taking it as an add-on therapy, but only at the six-month timepoint. Furthermore, the decrease in IL-1-beta was higher in the younger boys. Conversely, the H_2_O_2_ concentrations significantly decreased in patients treated with flavocoxid alone and at both the 6- and 12-month timepoints, associated with a secondary reduction of serum GPx levels. The reduction in H_2_O_2_ and GPx was more evident in the younger boys. On the one hand, these findings confirm that flavocoxid has anti-inflammatory and antioxidant activities in DMD boys as well, while on the other hand, it was able to exert anti-inflammatory effects only if combined with steroids, and for a limited period of time, but a definite antioxidant property when applied alone and for the entire treatment period. Both the anti-inflammatory and antioxidant activities were higher if the flavocoxid treatment was initiated at an earlier age, confirming what is known for steroids in younger DMD boys [43].

The secondary endpoint of this phase 1/2 study was the functional assessment of motor and respiratory functions, which are usually checked in the natural history and therapeutic studies of DMD [30,44]. We were unable to see positive effects in the flavocoxid-treated boys compared to the untreated group. A reason could be that a disease-modifying effect of flavocoxid would be found if started early in the disease course and given on a long-term basis. Indeed, a recent trial comparing three steroid regimens have been planned for a minimum of three years and a maximum of five years to assess the relative effectiveness in DMD [45] However, we found, in our pilot study, a trend showing that younger boys who received treatment were faster at performing the Gowers’ maneuver, and older boys who received treatment were better at doing 10MWT. A longer trial duration and a larger sample size could help to better evaluate the magnitude of these effects. Moreover, another limitation of this study was the contemporary therapy with steroids in the majority of patients, which might have masked the effects of flavocoxid. On the contrary, the diffuse utilization of corticosteroids as the standard of care for DMD makes it unethical to stop them. However, the effect size of the secondary outcomes in this phase 1/2 study can be used for power analysis in the design of future studies.

## 5. Conclusions

In conclusion, this first study of flavocoxid in DMD boys showed that it was well tolerated and able to induce a reduction in the serum biomarkers of inflammation response and oxidative stress, most likely through a pivotal NF-κB inhibition. Although the functional assessments, tested as secondary endpoints, did not show significant differences given the modest number of subjects, a further double-blind, placebo-controlled study for at least two/three years is warranted to verify its potential as a steroid substitute or as an add-on therapy to steroids. Flavocoxid could also be investigated to reduce steroid dosing in DMD.

## Figures and Tables

**Table 1:** Baseline characteristics of the Duchenne muscular dystrophy (DMD) patients, expressed as mean ± SD (range).

	Flavocoxid-Treated (*n* = 17)	Untreated (*n* = 17)	Significance
Age, years	6.5 ± 2.2 (4–12)	6.1 ± 2.4 (4–12)	n.s.
Steroid, dosage	prednisone, 0.7 mg/kg/alternate days (*n* = 2) deflazacort, 0.9 mg/kg/alternate days (*n* = 5) deflazacort, 0.9 mg/kg/day (*n* = 2)	prednisone, 0.7 mg/kg/alternate days (*n* = 3) deflazacort, 0.9 mg/kg/alternate days (*n* = 4) deflazacort, 0.9 mg/kg/day (*n* = 3)	
Steroid duration, months	26.4 ± 12.5 (8–46)	25 ± 19.3 (7–60)	n.s.
CK, U/L	11,490 ± 5010 (3122–20,011)	15,028 ± 6046 (3494–21,378)	n.s.
AST, U/L	261 ± 73 (120–365)	312 ± 109 (193–585)	n.s.
ALT, U/L	411 ± 187 (178–831)	428 ± 87 (295–575)	n.s.
LDH, U/L	1932 ± 836 (810–3380)	2158 ± 780 (1132–3075)	n.s.
NSAA	28.8 ± 6.25 (16–34)	27.2 ± 5.7 (16–34)	n.s.
Gowers’ time, s	8.59 ± 13.8 (3.1–60)	8.47 ± 13.5 (2.1–60)	n.s.
10MWT, s	6.1 ± 2.4 (3.8–13.1)	5.5 ± 1.68 (3–10)	n.s.
6MWT, m	387 ± 96.7 (175–521)	358 ± 55.6 (225–475)	n.s.
FVC, L	1.18 ± 0.56 (0.47–2.79)	1.11 ± 0.54 (0.63–2.78)	n.s.

s, seconds; m, meters; L, liters; n.s., not significant.

**Table 2:** Change in outcomes at T12 vs. T0 in the treated and untreated patients, all grouped together (*n* = 17 and 17, respectively) and divided according to age < 7 years (*n* = 11 and 11, respectively) and age > 7 years (*n* = 6 and 6, respectively). Data are expressed as mean ± SEM.

	Treated	Untreated		Treated < 7 Years	Untreated < 7 Years		Treated > 7 Years	Untreated > 7 Years	
Age, years	6.5 ± 0.54	6.1 ± 0.57	n.s.	5.1 ± 0.2	4.6 ± 0.2	n.s.	9.1 ± 0.7	8.7 ± 0.8	n.s.
NSAA	−3.2 ± 1.4	−1.8 ± 1.1	n.s.	−1.7 ± 1.7	−0.8 ± 1.9	n.s.	−6.0 ± 2.4	−3.1 ± 0.7	n.s.
Gowers’ time, s	4.5 ± 2.4	4.9 ± 2.7	n.s.	3.0 ± 1.5	6.9 ± 4.4	n.s.	7.2 ± 6.6	2.1 ± 1.6	n.s.
10MWT, s	0.6 ± 0.5	1.3 ± 0.7	n.s.	0.5 ± 0.5	0.5 ± 0.8	n.s.	0.8 ± 1	2.3 ± 1.3	n.s.
6MWT, m	−9.7 ± 14	−2.6 ± 20	n.s.	−2.1 ± 20	17 ± 28	n.s.	−24 ± 14	−25 ± 27	n.s.
FVC, L	0.07 ± 0.07	0.04 ± 0.06	n.s.	0.09 ± 0.06	0.05 ± 0.09	n.s.	0.03 ± 0.15	0.03 ± 0.06	n.s.

s, seconds; m, meters; L, liters; n.s., not significant.

## Data Availability

The data presented in this study are available on request from the corresponding author.
